# The impact of IgSF11-PKM2 pathway on gene expression in osteoclasts

**DOI:** 10.17912/micropub.biology.001469

**Published:** 2025-03-24

**Authors:** Hyunsoo Kim, Noriko Takegahara, Yongwon Choi

**Affiliations:** 1 Department of Pathology and Laboratory Medicine, University of Pennsylvania Perelman School of Medicine, Philadelphia, PA, USA

## Abstract

Osteoclasts are primary bone resorbing cells. Previously, we described metabolic regulation of osteoclasts through IgSF11-mediated phosphorylation of the glycolytic enzyme PKM2. Here, we report the impact of IgSF11-PKM2-mediated regulation on gene expression in osteoclasts, utilizing RNA sequencing on osteoclasts engineered to express a chimeric protein, lacking IgSF11, and pharmacologically modulating PKM2 activity. Our analysis identified osteoclast-related genes whose expression is altered by the absence of IgSF11 and by changes in PKM2 activity. This study reveals gene expression changes associated with the IgSF11-PKM2 pathway, providing new insight into its role in osteoclasts.

**Figure 1. RNA sequencing analysis identifies the impact of the IgSF11-PKM2 pathway on gene expression in osteoclasts:**
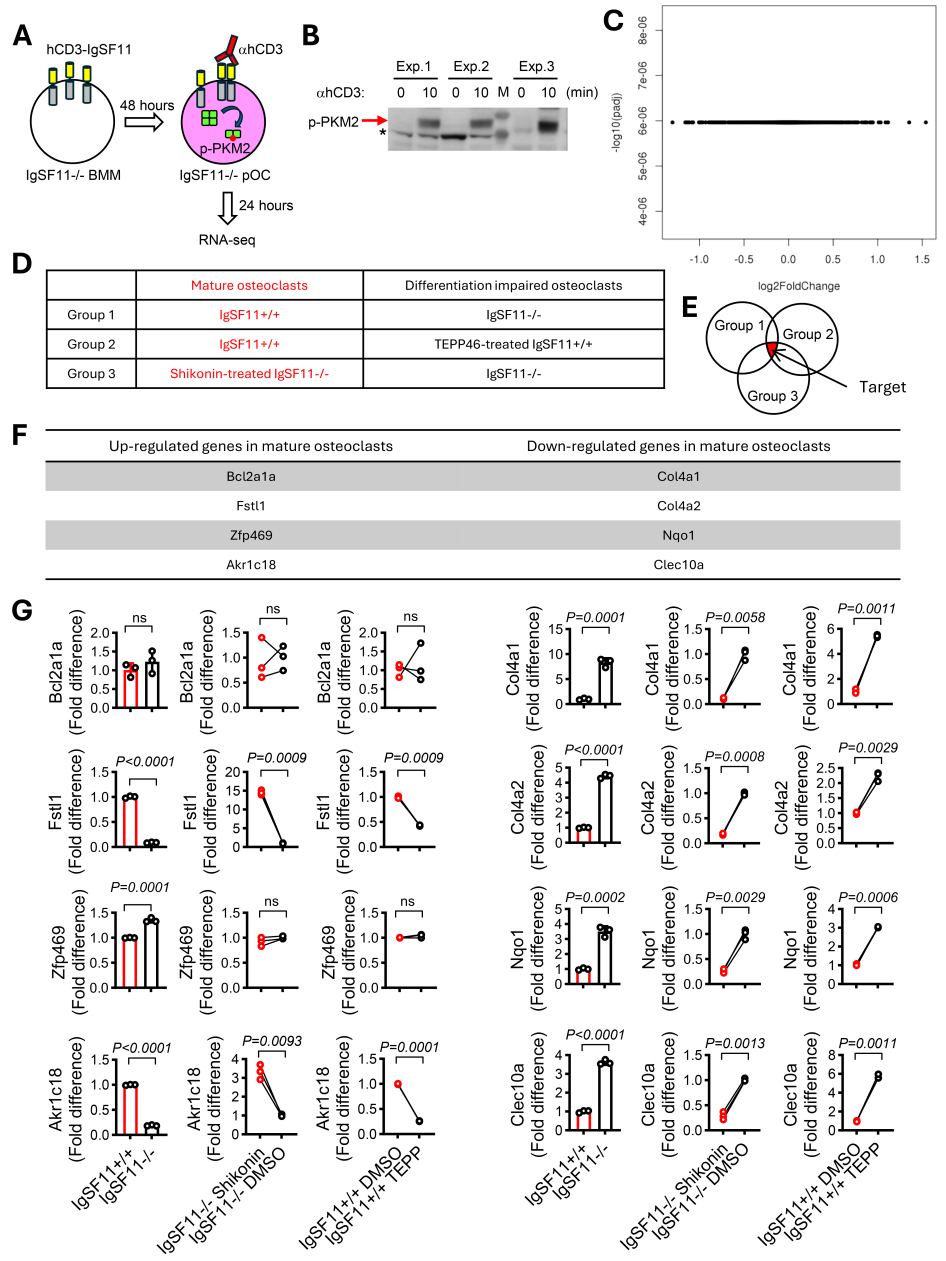
**A.** Schematic diagram of experimental design. BMMs from IgSF11 ^−/−^ mice were retrovirally transduced with an hCD3-IgSF11 expression vector. The cells were cultured with M-CSF and RANKL for 48 hours to generated pre-osteoclasts. Then, the cells were stimulated with anti-hCD3 antibodies for 24 hours to cross-link surface hCD3 and stimulate the IgSF11 intracellular region in the presence of M-CSF and RANKL. Isotype-matched antibodies were used as a control. Three biological replicates of samples were prepared. Total RNA was collected from the cells, and RNA-seq analysis was performed. **B.** Confirmation of functionality of the system. Three biological replicates of hCD3-IgSF11 transduced cells were prepared, stimulated with anti-hCD3 antibodies for 10 min, and the phosphorylation of PKM2 was examined by western blotting. Red arrow indicates phosphorylated PKM2. Asterisk indicates non-specific bands. M: Protein size marker. **C.** Volcano plot of differentially expressed genes between cells stimulated with anti-hCD3 antibodies and those with control isotype antibodies. **D.** Three experimental groups of mature osteoclasts and differentiation-impaired osteoclasts. **E.** Venn diagram depicting the experimental schematic. Overlapping genes among the three experimental groups were identified as IgSF11-PKM2-regulated genes in osteoclasts. **F.** Eight genes identified as significantly differentially expressed and associated with IgSF11-PKM2 regulation in osteoclasts. Four genes were significantly upregulated in mature osteoclasts, while four others were significantly downregulated in mature osteoclasts. **G.** Validation of the expression profiles of the eight genes. Three biological replicates of IgSF11 ^+/+^ and IgSF11 ^−/−^ BMMs were cultured with M-CSF + RANKL to induce osteoclasts in the presence of shikonin or TEPP46 for three days. Total RNA was isolated from the cells for qPCR analysis. IgSF11 ^+/+^ and IgSF11 ^-/-^ osteoclasts are independent samples, and the results are shown as bar graphs, with a two-tailed unpaired Student’s *t* test performed. IgSF11 ^-/-^ cells treated with or without Shikonin, and IgSF11 ^+/+^ cells treated with or without TEPP46 are paired samples. These results are shown as line graphs, with a two-tailed paired Student’s *t* test performed. Each line represents paired samples. Validation of the upregulated genes confirmed significant upregulation of *Fstl1* and *Akr1c18* , while validation of the downregulated genes confirmed significant downregulation of *Col4a1* , *Col4a2* , *Nqo1* , and *Clec10a* . Mature osteoclasts are labeled in red, while osteoclasts with impaired differentiation are labeled in black.

## Description

Osteoclasts are bone-resorbing, multinucleated giant cells whose differentiation and function are regulated in a highly intricate manner (Takegahara, Kim, & Choi, 2024). Osteoclast differentiation requires the engagement of costimulatory molecules, along with the osteoclast differentiation factor receptor-activated NF-kB ligand (RANKL), to establish not only cell-cell contacts but also mediate intracellular signal transduction for optimal activation and differentiation (Humphrey & Nakamura, 2016). In addition, to meet the demands of energy production and anabolic processes, osteoclasts require the glycolytic pathway to oxidize glucose, directing a fraction of glucose metabolites into anabolic pathways that contribute to macromolecule synthesis, such as protein, lipid, and nucleic acid (Hyunsoo Kim, Takegahara, & Choi, 2023). We previously reported that immunoglobulin superfamily member 11 (IgSF11), a member of the immunoglobulin superfamily, engages in homophilic interactions to promote osteoclast differentiation by regulating the activity of pyruvate kinase isoform M2 (PKM2), a key enzyme in glycolysis (Hyunsoo Kim et al., 2023; H. Kim et al., 2020). This regulation facilitates the production of glycolytic intermediates during late phase of osteoclast differentiation. PKM2’s role in the metabolic regulation of osteoclasts is further supported by observations that activation of PKM2 with the specific activator TEPP46 reduces glycolysis and inhibits the formation of large multinucleated osteoclasts, whereas PKM2 inhibition with the specific inhibitor shikonin enhances osteoclast differentiation (Hyunsoo Kim et al., 2023). Beyond its cytoplasmic role in regulating glycolysis, PKM2 exhibits non-glycolytic functions in the nucleus. The PKM2 protein consists of four structural units. While tetrameric PKM2 predominantly resides in the cytoplasm and exerts glycolytic function, dimeric PKM2, which displays low catalytic activity and provides glycolytic intermediates for anabolism, often translocates into the nucleus, where it regulates transcription factors and influences various signaling pathways (Wong, Ojo, Yan, & Tang, 2015; Zhang et al., 2019). Given that PKM2 influences gene expression, this research aims to investigate whether the IgSF11-PKM2 pathway regulates gene expression in osteoclasts.

First, we investigated whether IgSF11-PKM2 signaling directly induces gene expression by employing a previously established system that specifically activates the cytoplasmic region of IgSF11 in a controlled manner using an hCD3-IgSF11 chimeric protein ( Figure 1A ) (Hyunsoo Kim et al., 2023). Bone marrow monocytes (BMMs) from IgSF11 ^−/−^ mice were retrovirally transduced with hCD3-IgSF11 and then cultured with RANKL for 48 hours to generate hCD3-IgSF11-expressing pre-osteoclasts. These cells were stimulated with anti-hCD3 antibodies for 24 hours to cross-link surface hCD3 and activate the intracellular region of IgSF11. Subsequently, total RNA was collected from the cells, and RNA-seq analysis was performed. In parallel, the cells were stimulated with anti-hCD3 antibodies for 10 min, and the tyrosine phosphorylation of PKM2 was examined by western blotting. Stimulation with anti-hCD3 antibodies resulted in the phosphorylation of PKM2, confirming that anti-hCD3 antibodies successfully activated the IgSF11 intracellular regions, leading to activation of PKM2 in the cells ( Figure 1B ). RNA-seq analysis revealed that none of the genes were significantly expressed following stimulation with anti-hCD3 antibodies ( Figure 1C ). These findings suggest that direct activation of the IgSF11 intracellular region, followed by PKM2 signaling through cross-linking of surface hCD3 with anti-hCD3 antibodies, is unlikely to induce gene expression.

Next, we prepared mature osteoclasts and differentiation-impaired osteoclasts using cells lacking IgSF11 and pharmacologically modulating PKM2 activity, then assessed gene expression differences between them. Mature osteoclasts include IgSF11 ^+/+^ osteoclasts and IgSF11 ^−/−^ osteoclasts treated with shikonin, while differentiation-impaired osteoclasts include IgSF11 ^+/+^ osteoclasts treated with TEPP-46 and IgSF11 ^−/−^ osteoclasts ( Figure 1D ). We established three experimental groups: Group 1: IgSF11 ^+/+^ osteoclasts and IgSF11 ^−/−^ osteoclasts, to reveal IgSF11-mediated changes in osteoclasts, including PKM2-related and unrelated changes; Group 2: IgSF11 ^+/+^ osteoclasts and IgSF11 ^+/+^ osteoclasts treated with TEPP-46, to identify PKM2-mediated changes in osteoclasts; Group 3: IgSF11 ^−/−^ osteoclasts treated with shikonin and IgSF11 ^−/−^ osteoclasts, to identify PKM2-mediated changes that rescue IgSF11 deficiency in osteoclasts ( Figure 1D ). Overlapping genes among the three experimental groups are assumed to represent IgSF11-PKM2-associated genes in osteoclasts ( Figure 1E ). This experimental condition was intended to identify genes regulated by IgSF11 and PKM2. IgSF11 ^+/+^ and IgSF11 ^−/−^ BMMs were induced to form osteoclasts in the presence or absence of TEPP-46 and/or shikonin, followed by isolation of total RNA. RNA-seq and differential gene expression analysis were conducted, identifying significantly differentially expressed genes in each group. Ultimately, eight genes were identified as significantly differentially expressed and related to IgSF11-PKM2 regulation in osteoclasts ( Figure 1 F). *Bcl2a1a* , *Fstl1* , *Zfp469* , and *Akr1c18* were upregulated, while *Col4a1* , *Col4a2* , *Nqo1* , and *Clec10a* were downregulated in mature osteoclasts. To validate these findings, new RNA samples were prepared, and gene expression levels were confirmed using qPCR. We observed significantly increased expression levels of *Fstl1* and *Akr1c18* and significantly decreased expression levels of *Col4a1* , *Col4a2* , *Nqo1* , and *Clec10a* , confirming six genes as significantly differentially expressed and associated with IgSF11-PKM2 regulation in osteoclasts ( Figure 1G ). Notably, as none of these genes were detected using the system with the hCD3-IgSF11 chimeric protein ( Figure 1C ), this suggests that their expression is altered by IgSF11 and PKM2 activation. Among these genes, *Fstl1* and *Nqo1* are reported to positively and negatively regulate osteoclast differentiation, respectively (Kanzaki, Shinohara, Kajiya, & Kodama, 2013; H. J. Kim et al., 2016; Wang et al., 2023), and *Akr1c18* is also implicated in osteoclasts (Guerit et al., 2020). Furthermore, *Fstl1* and *Nqo1* are known to be linked to PKM2 (Rao et al., 2022; Zhu et al., 2020). Taken together, our results identify six genes as IgSF11-PKM2-associated, osteoclast-related genes. This study provides new insights into the role of IgSF11-PKM2 beyond its function as a metabolic regulator in osteoclasts.

## Methods

**Mice**

IgSF11-knockout (IgSF11 ^−/−^ ) mice were generated as described previously (H. Kim et al., 2020). Homozygous IgSF11 ^+/+^ and IgSF11 ^−/−^ littermate mice were generated by intercrossing heterozygous mice. C57BL/6 mice were purchased from the Jackson Laboratory. All experimental procedures were performed in accordance with the guidelines approved by the Institutional Animal Care and Use Committee (IACUC) of the University of Pennsylvania.

**Cell Culture**

Cells were prepared as described previously (H. Kim et al., 2020). Briefly, BMMs were induced from whole bone marrow cells cultured with macrophage colony-stimulating factor (M-CSF) in a-MEM containing 10% FCS for three days. Pre-osteoclasts and osteoclasts were induced from BMMs with M-CSF (60 ng/ml) + RANKL (150 ng/ml) for two days and three days, respectively. TEPP46 (Cat.# ML-265) and Shikonin (Cat.# 14751-10) were purchased from Cayman Chemical. Anti-human CD3 (HIT3a, Cat.# 300302) antibodies and isotype antibodies (Cat.# 401502) were purchased from Biolegend. Antibodies were used at 1 mg/ml, TEPP46 was uses at 2 mg/ml, and shikonin was used at 40 ng/ml.

**Retrovirus preparation and transduction**

Virus particle preparation and transduction were performed as described previously (Hyunsoo Kim et al., 2023). Plat-E packaging cells were transfected with pMX vectors encoding C-terminally FLAG-tagged hCD3-iFL using PEImax (Polysciences). After three days, medium containing each retrovirus was harvested and passed through a syringe filter (0.45 mm pore diameter). BMMs were transduced with retroviruses for 16 hours with hexadimethrine bromide (8 mg/ml) in the presence of M-CSF (60 ng/ml). After washing with fresh medium, infected cells were selected by culturing for 2 days in the presence of puromycin (2 mg/ml) and M-CSF (60 ng/ml). Puromycin-resistant BMMs were harvested and re-seeded into a 6-well plate at a density of 1 × 10⁶ cells per well. For RNA sequencing, adherent cells were stimulated with anti-hCD3 antibodies (1 µg/mL) or isotype antibodies for 24 hours. To confirm PKM2 phosphorylation, cells were stimulated with anti-hCD3 antibodies (1 µg/mL) for 10 minutes.

**Western blot analysis**

Cell cultures were lysed with ice-cold RIPA lysis and extraction buffer (Cat.# 89900; Thermo Fisher) containing a protease and phosphatase inhibitor cocktail (Roche). The protein concentrations were determined using a Bradford assay, followed by electrophoretic resolution and transfer to PVDF membranes. Western blot was performed with the anti-phospho PKM2 Y105 (Cat.# 3827; Cell Signaling Technology).

**RNA sequencing**

Total RNA was isolated from cultured cells using RNeasy Plus Mini (QIAGEN). The total RNA samples were shipped to Azenta Life Sciences in dry ice, and cDNA library preparation, RNA sequencing and data processing were performed by Azenta Life Sciences. The Standard RNA sequencing service prepared the library for coding and long non-coding RNA. mRNA was isolated using poly(A) selection. Sequencing was conducted on the Illumina platform under the following conditions: a 2 × 150 base pairs configuration, a read depth of 20 million, and a guaranteed data quality of ≥80% bases with at least Q30. Sequence reads were trimmed to remove possible adapter sequences, and mapped to the Mus musculus GRCm38 reference genome available on ENSEMBL. Unique gene hit counts were calculated. After extracting gene hit counts, gene expression was compared between anti-hCD3 stimulated and isotype antibody stimulated groups using DESeq2. The Wald test was used to generate *p-* values and log2 fold changes. Genes with an adjusted *p* -value < 0.05 and an absolute log2 fold change > 1 were classified as differentially expressed.

**Reverse transcription and qPCR**

Total RNA was extracted from cells using RNeasy Plus Mini (QIAGEN), and 1mg–5 mg of total RNA was reverse transcribed using random hexamer primers and SuperScript III reverse transcriptase (Invitrogen). cDNA corresponding to 10 ng of total RNA was analyzed by Q-PCR using a QuantStudio3 (Applied Biosystems) and the following specific TaqMan probes: Bcl2a1a (Mm00833201_s1), Fstl1 (Mm00433371_m1), Zfp469 (Mm00626403_m1), Akr1c18 (Mm00506289_m1), Col4a1 (Mm01210125_m1), Col4a2 (Mm00802386_m1), Nqo1 (Mm01253561_m1), Clec10a (Mm00546125_g1), and 18 S (Hs99999901_s1). The CT method of relative quantification was used to determine the fold change in expression.

**Statistical analysis**

Two-tailed unpaired and paired Student’s *t* test were used to determine the significance of differences by Prism 10.4.1 (GraphPad Software). *P* < 0.05 was considered statistically significant.
